# Supplementary material for: The Arabidopsis Hypoxia Inducible AtR8 Long Non-Coding RNA also Contributes to Plant Defense and Root Elongation Coordinating with WRKY Genes under Low Levels of Salicylic Acid
Source: Noncoding RNA. 2020 Feb 26;6(1):8. doi: 10.3390/ncrna6010008 (PMC7151572; doi:10.3390/ncrna6010008)
Supplement: Supplementary file 1 [file ncrna-06-00008-s001.zip › Supplemental/Supplemental Fig..pdf]

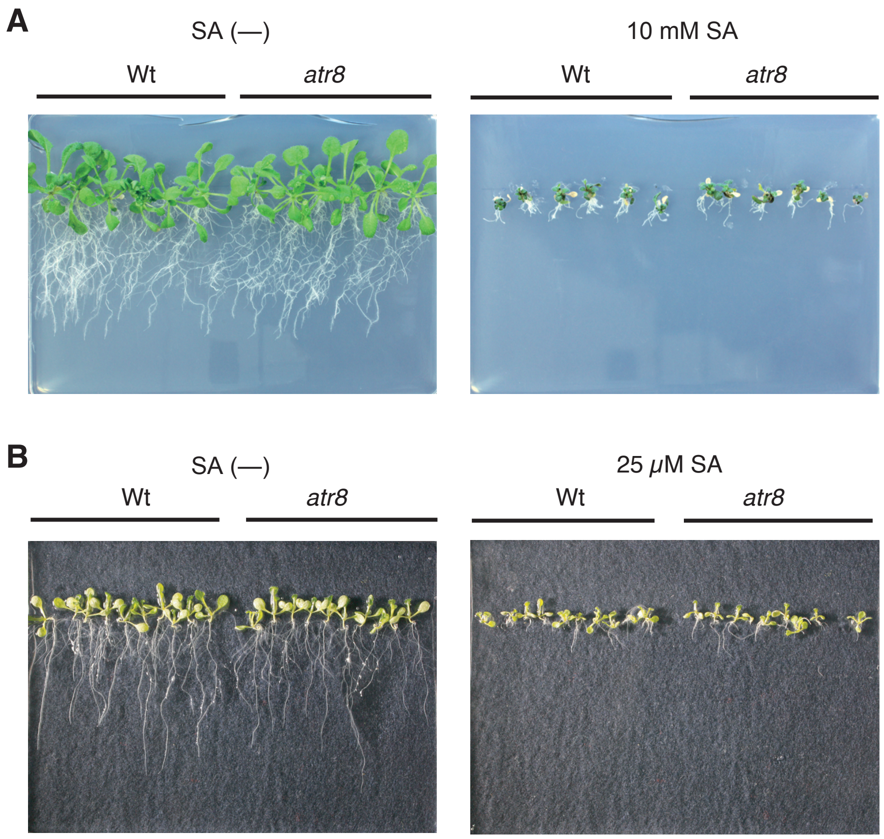

Supplemental Fig. S1. Inhibitory effects of root elongation under different SA conditions. Root lengths of Arabidopsis seedling (Wt and *atr8*) were compare with different SA conditions. (A) Seedling were germimated on horizontal growth media without SA, and transplanted to 10 mM SA at 9 days after germination, and grown for 13 days on vertical plane of medium with or without 10 mM SA (including 1.2% (w/v) agar, 1/2 MS and 2% (w/v) sucrose). (B) Seedling were germimated on vertical growth media with or without 25 μM SA (including 0.24% (w/v) gellan gum, 1/2 MS and 2% (w/v) sucrose), and then grown for 14 days. Any clear difference was not observed between Wt and *atr8* mutant under 10 mM and 25 μM SA conditions.

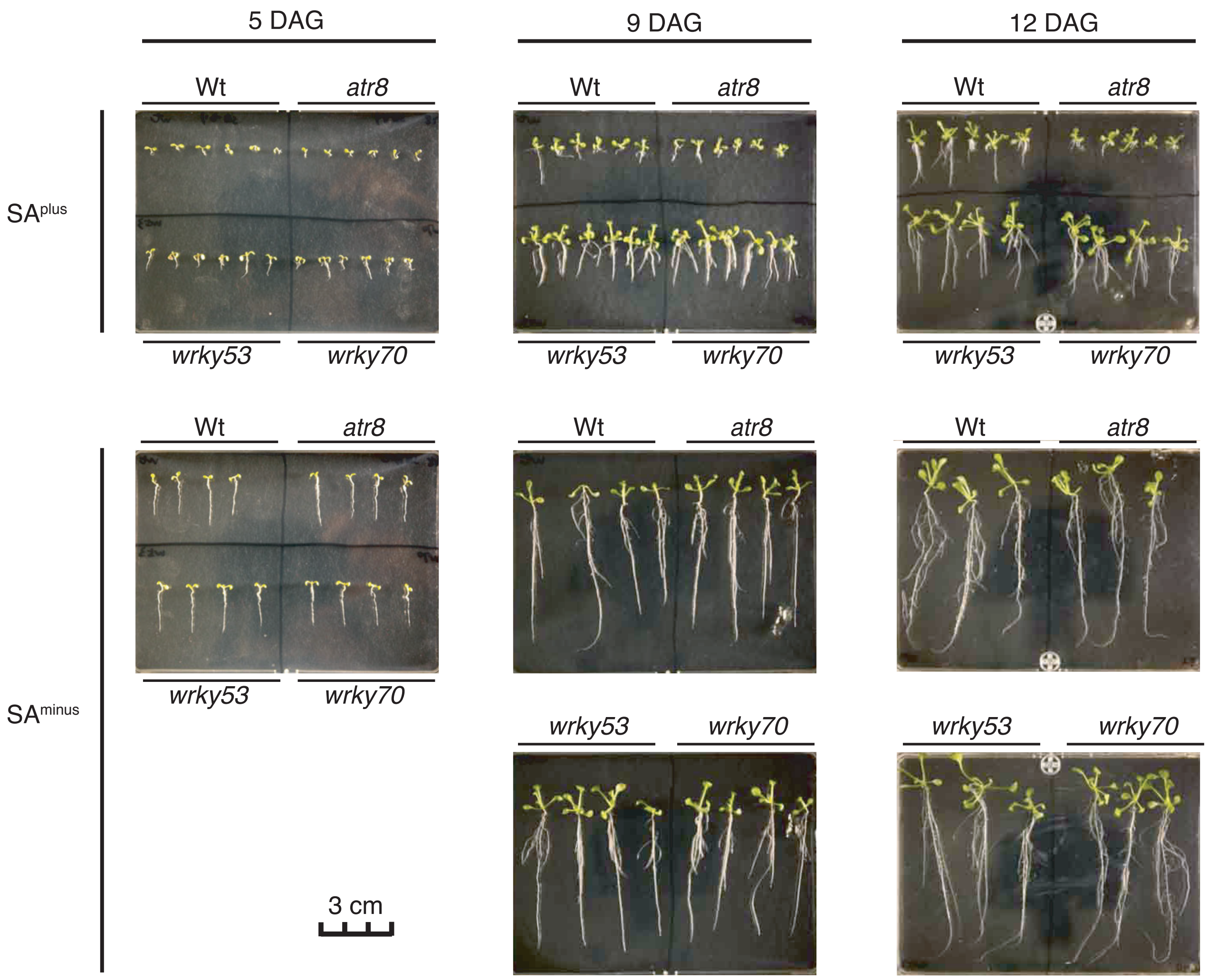

Supplemental Fig. S2. Inhibitory effects of root elongation with 20 μM SA condition. Root lengths of Arabidopsis seedling were compared, which were grown on vertical 0.24% gellan gum media with (SA^{plus}) or without (SA^{minus}) 20 μM salicylic acid for 5, 9, 12 days after germination (DAG).

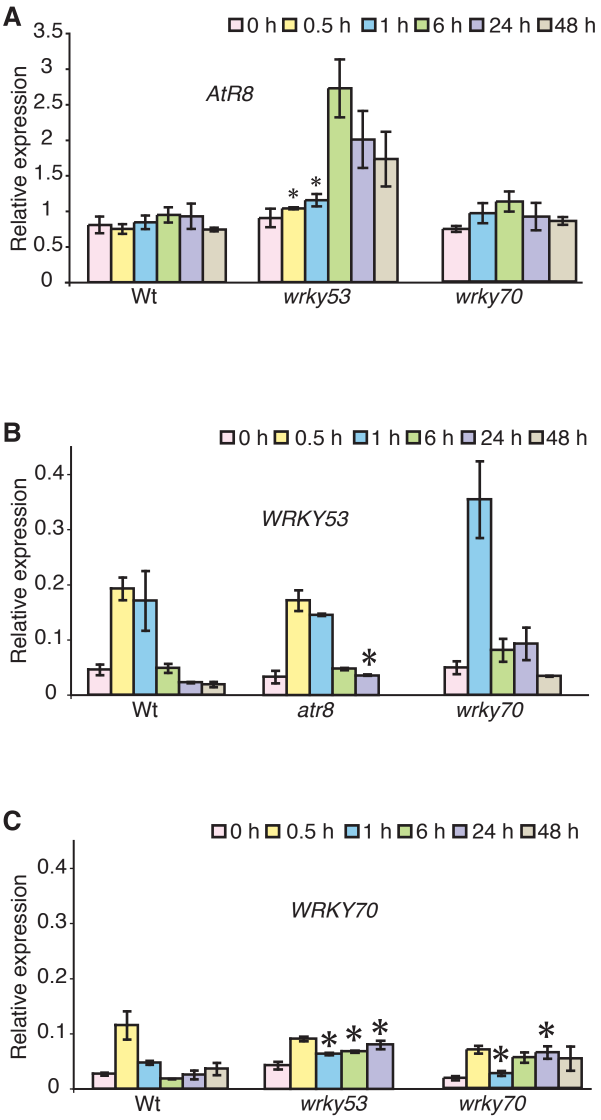

Supplemental Fig. S3. Short-term time course of RNA levels induced by infection with *Pseudomonas syringae*. Expression levels of *AtR8* lncRNA as well as *WRKY53* and *WRKY70* in Arabidopsis plants treated with Ps, which were cultured with 500 μL of King´s liquid media and suspended in 50 mL of sterile water (100 times dilution). Gene expression levels with Ps or water control were compared by RT-qPCR. Bar graphs represent mean values of three independent assays (≥50 plants), and error bars represent ±SE. (A): *AtR8*, (B): *WRKY53*, and (C): *WRKY70*. Significant differences in RNA accumulations levels between Wt and each of the mutants by (Welch´s *t*-test) are indicated as *$p < 0.05$.

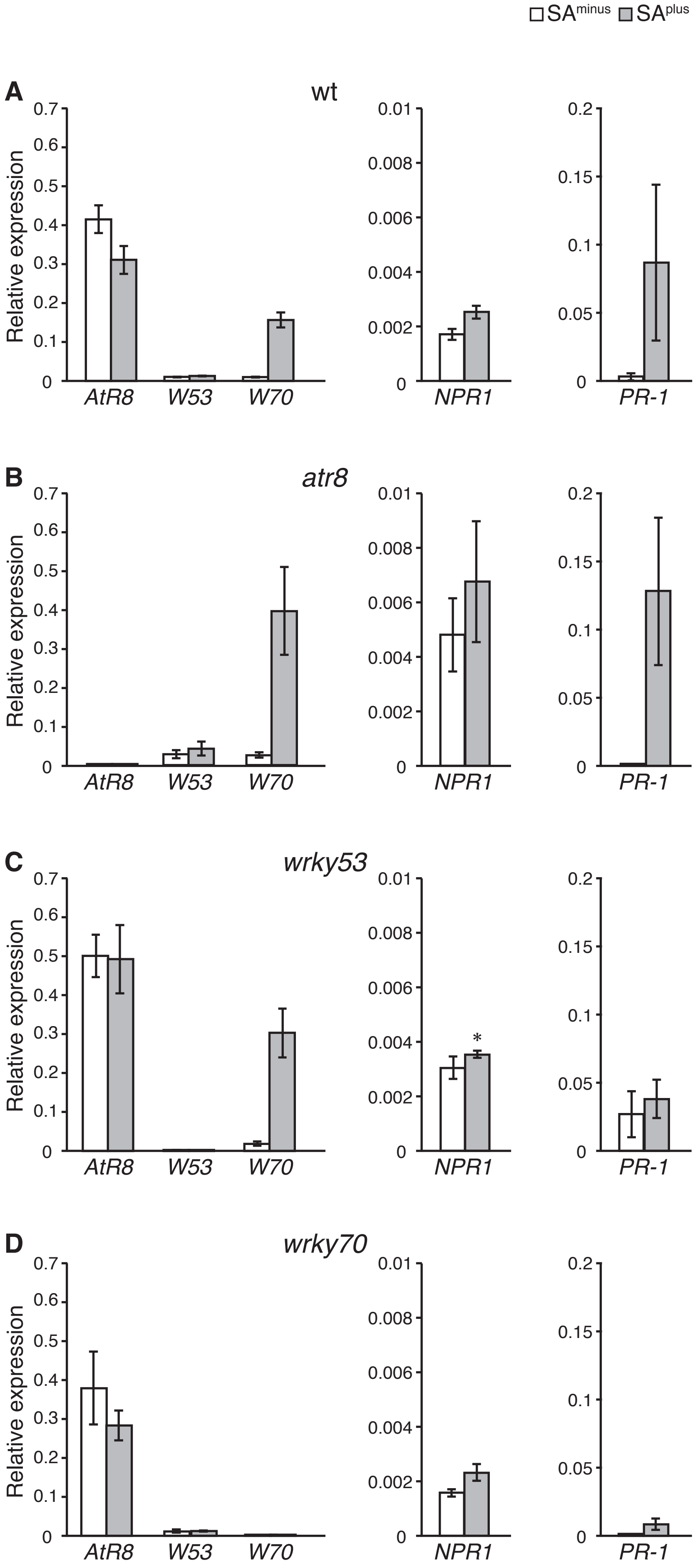

Supplemental Fig. S4. Expression levels of RNAs in Arabidopsis roots under continuous treatment with 20 μM SA. Total RNAs were extracted from roots, 24 days after seeding (≥ 40 plants). Gene expression levels with (SA^{plus}) or without (SA^{minus}) SA were compared by RT-qPCR. Bar graphs represent mean values of three independent assays, and error bars represent ±SE. *W53*: *WRKY53*, *W70*: *WRKY70*. (A): Wt, (B): *atr8*, (C): *wrky53*, and (D): *wrky70*. Significant differences in RNA accumulation levels between Wt and each of the mutants (Welch´s *t*-test) are indicated as $*p < 0.05$.
